# Supplementary material for: Impact of Microbiota Diversity on Inflammatory Bowel Disease
Source: Microorganisms. 2025 Mar 21;13(4):710. doi: 10.3390/microorganisms13040710 (PMC12029714; doi:10.3390/microorganisms13040710)
Supplement: Supplementary file 1 [file microorganisms-13-00710-s001.zip › Figures S1-S4.pdf]

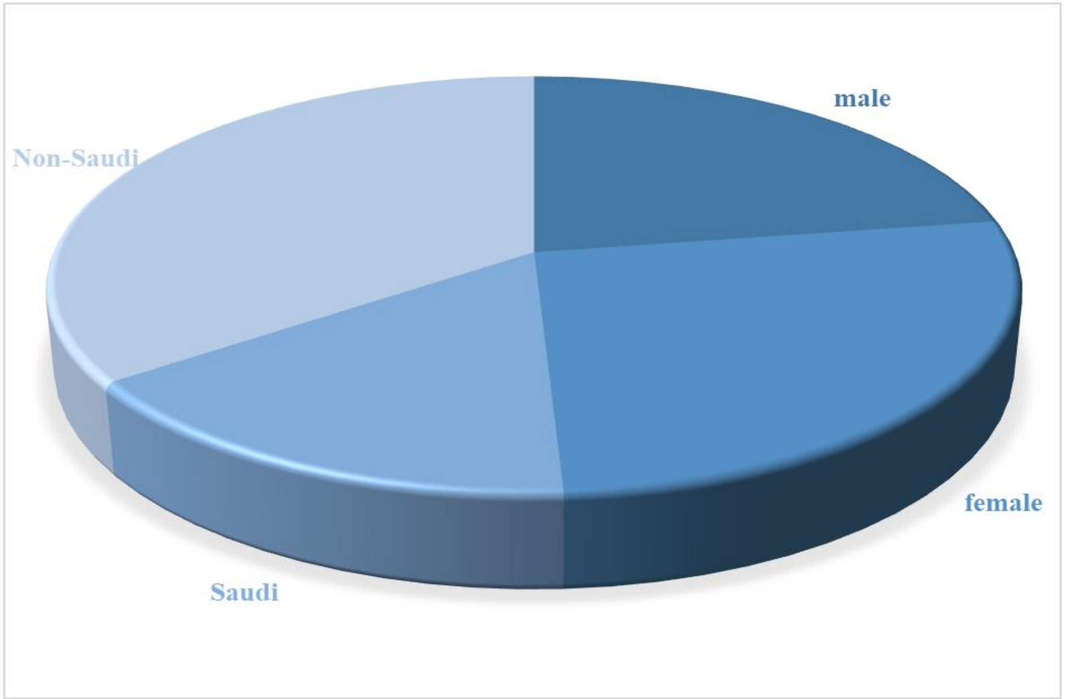

Figure S1. The distribution of study participants who's registered in selected hospitals in Makkah by gender and nationality.

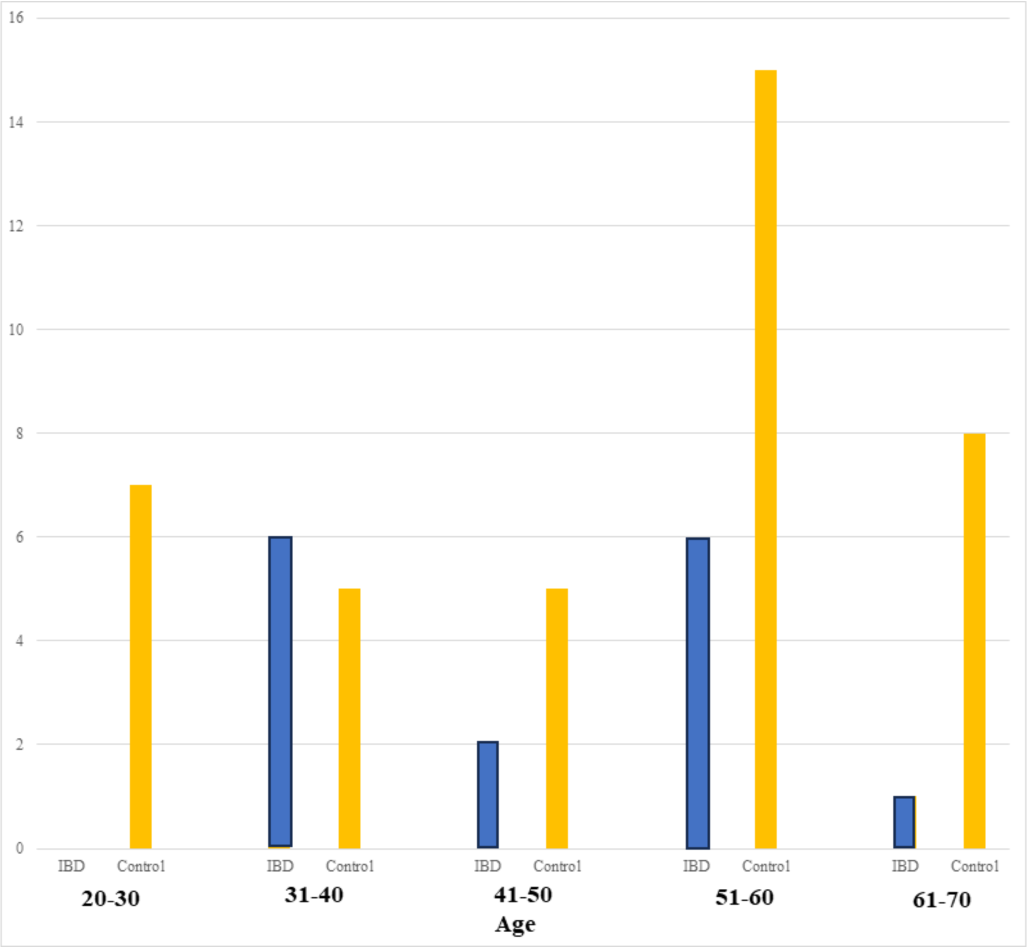

Figure S2. The distribution of study participants who's registered in selected hospitals in Makkah by age.

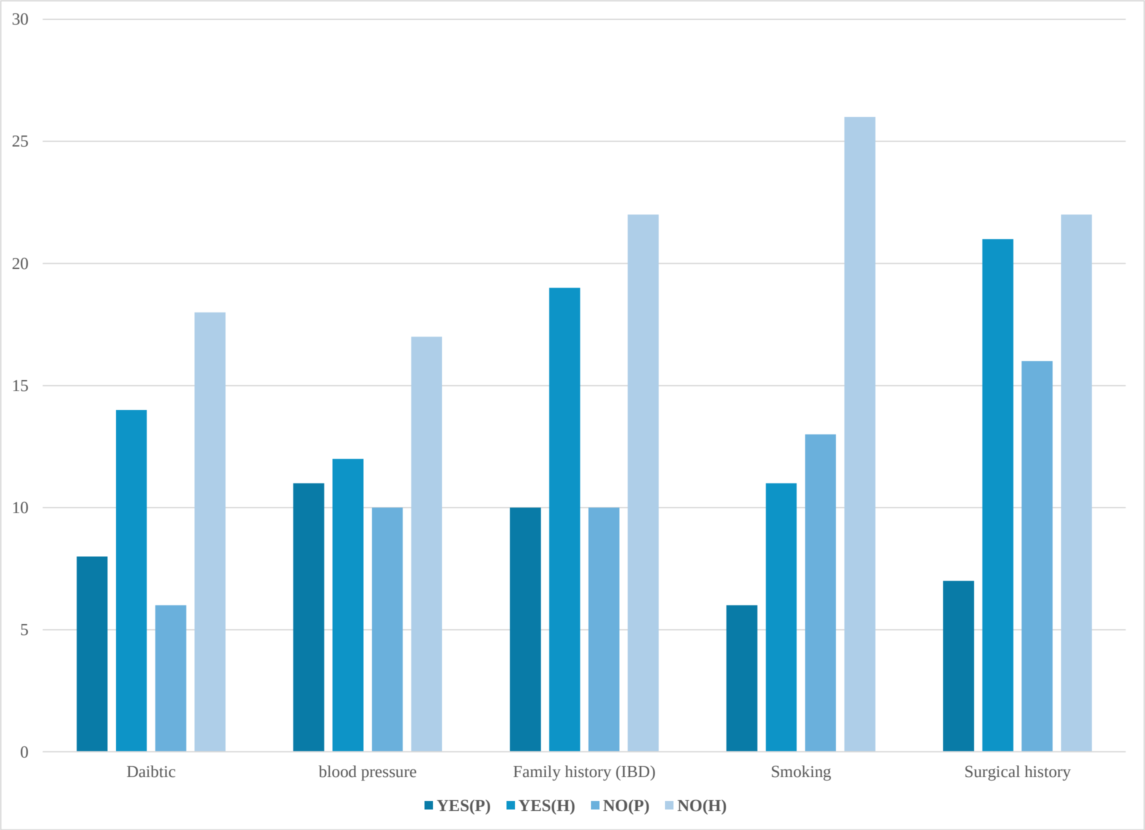

Figure S3. Medical history of study participants who’s registered in selected hospitals in Makkah.

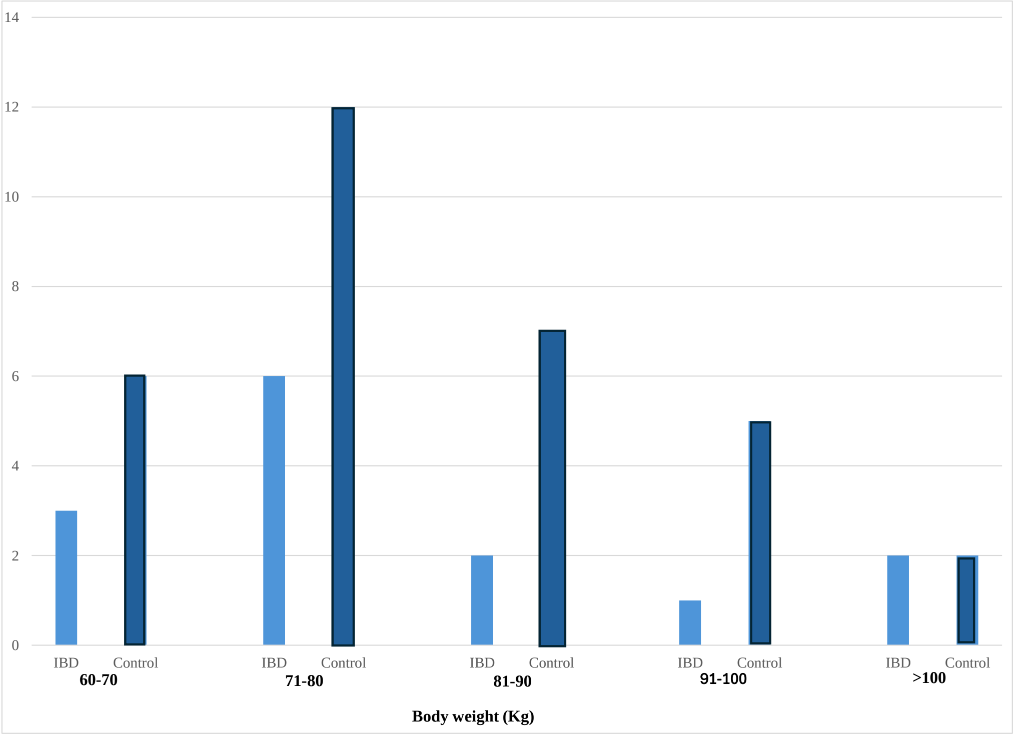

Figure S4. Body weight (Kg) of study participants who's registered in selected hospitals in Makkah.
